# Associations Between Diabetes-Specific Disordered Eating Behaviors, Social Anxiety, Social Appearance Anxiety, and Psychological Resilience in Adolescents with Type 1 Diabetes

**DOI:** 10.3390/children13060732

**Published:** 2026-05-25

**Authors:** Ayse Nur Durmus, Havva Akpınar

**Affiliations:** 1 Department of Psychiatric Nursing, Institute of Health Sciences, Mugla Sıtkı Kocman University, Mugla 48000, Türkiye; anurdurmus422@gmail.com; 2 Department of Psychiatric Nursing, Faculty of Health Sciences, Mugla Sıtkı Kocman University, Mugla 48000, Türkiye

**Keywords:** type 1 diabetes, adolescents, disordered eating, social anxiety, social appearance anxiety, psychological resilience

## Abstract

**Highlights:**

**What are the main findings?**

- A substantial proportion of adolescents with type 1 diabetes exhibited elevated levels of diabetes-specific disordered eating behaviors.
- Social anxiety and social appearance anxiety showed a significant independent association with diabetes-specific disordered eating behaviors, whereas psychological resilience was inversely related to both social anxiety and social appearance anxiety.

**What are the implications of the main findings?**

- Routine psychosocial screening for diabetes-specific disordered eating behaviors and anxiety symptoms should be integrated into adolescent diabetes care.
- Interventions targeting social anxiety and social appearance concerns may be beneficial, while strengthening psychological resilience may support overall psychosocial well-being.

**Abstract:**

**Background**: Adolescents with type 1 diabetes (T1D) face considerable psychosocial demands that may increase vulnerability to diabetes-specific disordered eating behaviors. This study investigated the relationships among diabetes-specific disordered eating behaviors, social anxiety, social appearance anxiety, and psychological resilience in adolescents with T1D. **Methods**: This cross-sectional and correlational study included 176 adolescents diagnosed with T1D. Data were obtained using the Diabetes Eating Problem Survey-Revised (DEPS-R), the Social Anxiety Scale for Adolescents (SAS-A), the Social Appearance Anxiety Scale (SAAS), and the Child and Youth Resilience Measure (CYRM-12). **Results**: The mean age was 14.16 ± 2.73 years; 51.1% were male, and 63.1% had a disease duration of ≥3 years. Elevated levels of diabetes-specific disordered eating behaviors were observed in 85.8% of participants. Mean scores were 36.06 ± 15.26 (DEPS-R), 58.86 ± 12.90 (SAS-A), 48.82 ± 12.09 (SAAS), and 35.17 ± 10.61 (CYRM-12). Disordered eating behaviors showed positive correlations with social anxiety and social appearance anxiety and negative correlations with psychological resilience (all *p* < 0.001). Regression analyses indicated that social anxiety remained independently associated with disordered eating behaviors, whereas social appearance anxiety and psychological resilience did not. Psychological resilience was inversely related to both anxiety measures. **Conclusions**: Diabetes-specific disordered eating behaviors are common in adolescents with T1D and are closely linked to social anxiety-related factors. Social anxiety appears to be a key associated variable. Although psychological resilience was not independently related to disordered eating behaviors, it showed inverse associations with social anxiety. These findings support integrating routine psychosocial screening and targeted interventions into multidisciplinary diabetes care.

## 1. Introduction

Diabetes mellitus (DM) is a chronic metabolic condition marked by persistent hyperglycemia, arising from impairments in insulin secretion, insulin action, or both. Over time, it can lead to significant complications affecting the cardiovascular system, eyes, kidneys, and nervous system. Owing to its rising prevalence, DM is considered a major global public health issue [1]. The disease is generally categorized into type 1 and type 2 diabetes. Type 1 diabetes (T1D), which commonly emerges during childhood or adolescence, requires lifelong insulin administration for effective management [2,3].

In type 1 diabetes (T1D), the irreversible loss of pancreatic beta cells requires continuous, lifelong management of the disease. Treatment primarily aims to achieve optimal metabolic regulation, reduce the risk of both acute and long-term complications, and enhance quality of life [2]. Within this framework, attention to psychosocial adjustment is as important as maintaining physiological stability. Managing T1D during adolescence may significantly influence psychological well-being and social development [4].

Individuals diagnosed with T1D are more likely to experience disordered eating behaviors. Such behaviors may include binge eating, restrictive intake, intentional omission of insulin doses, self-induced vomiting, and excessive physical activity [5,6]. The term “diabulimia,” although not formally included in the Diagnostic and Statistical Manual of Mental Disorders (DSM-5), describes a form of disordered eating in which individuals intentionally restrict or omit insulin to affect body weight, often accompanied by compensatory behaviors such as self-induced vomiting, excessive physical activity, or the use of laxatives. These behaviors are associated with poor metabolic outcomes and may pose serious health risks. Additional features may include irregular attendance at clinical appointments, denial of weight-related concerns, heightened preoccupation with body image, preference for restrictive dietary patterns, and persistent monitoring of caloric intake. Diabulimia is reported more frequently during adolescence and may present greater challenges in disease management [6,7,8].

People with T1D must adjust their eating habits and lifestyle throughout their lives to keep their blood sugar levels in check. The need to follow specific dietary plans, the presence of restricted foods, and living with a chronic condition can cause anxiety in these individuals [8]. In addition, particularly during adolescence, issues such as body image, excessive concern with physical appearance, and weight problems arise. These circumstances disrupt the social relationships of adolescents with T1D, making them more introverted, weakening social interaction and communication, and causing them to experience negative feelings and thoughts about their bodies. Along with social anxiety, social appearance anxiety also emerges because of individuals’ negative body image regarding their body and appearance [7,9,10].

T1D, particularly during adolescence, is associated not only with biological and physical challenges related to the disease but also with a range of psychological and social difficulties. In this context, a biopsychosocial approach plays a critical role in ensuring effective disease management [11]. The biopsychosocial model provides an integrative perspective on health and illness by considering the interplay of biological, psychological, and social factors. Moving beyond a purely biomedical perspective, this approach facilitates a more holistic evaluation of individuals’ psychological and social circumstances, acknowledging the dynamic interplay among these factors. Originally proposed by Engel (1977) [12], the model highlights the importance of addressing biological, psychological, and social components together to achieve effective clinical care. 

In adolescents with T1D, disease-related demands (e.g., insulin management and dietary regulation) interact with psychosocial stressors such as peer relationships, body image concerns, and sensitivity to social evaluation, potentially increasing vulnerability to maladaptive eating behaviors [10]. From a biopsychosocial perspective, examining diabetes-specific disordered eating behaviors together with social anxiety, social appearance anxiety, and psychological resilience is expected to provide a comprehensive framework for understanding these complex interactions.

Previous research has identified meaningful links between disordered eating risk and psychosocial factors in individuals with T1D. A recent meta-analysis highlighted strong associations between eating-related problems and behaviors such as binge eating and bulimic tendencies in this population [10]. In adolescents with T1D, better sleep quality has been associated with higher levels of psychological resilience and improved quality of life [11]. Moreover, children and adolescents with T1D tend to report elevated levels of anxiety and depression, reduced quality of life, and difficulties in emotional regulation [13]. Collectively, these findings emphasize the need to consider not only the physiological aspects of the disease but also its psychological implications. Within this perspective, psychological resilience can be understood as the capacity to adapt to stressful circumstances and effectively cope with adversity [14].

Individuals with higher levels of resilience tend to manage chronic illness more effectively and report lower levels of psychological distress [15]. Research on adolescents with T1D suggests that resilience is positively related to quality of life and psychosocial adaptation [11]. From a stress–coping perspective, adolescents with T1D are exposed to chronic stressors that require continuous self-regulation and adaptation. Difficulties in coping with disease-related stress and negative emotions, or the use of ineffective coping strategies, may complicate disease management. In contrast, the use of effective coping strategies has been associated with better adaptation to diabetes-related demands and improved disease management [16].

Accordingly, psychological resilience can be conceptualized as an adaptive capacity linked to more effective coping and more favorable psychosocial outcomes. Greater resilience has been linked to reduced psychological distress and improved adjustment among adolescents with T1D [11]. Therefore, considering psychological resilience together with diabetes-specific disordered eating behaviors and social anxiety may offer a more comprehensive perspective on psychosocial functioning in this population.

Given that diabetes-specific disordered eating behaviors, social anxiety, and social appearance anxiety frequently co-occur in adolescents with T1D, examining these variables within an integrated framework is essential. However, studies that address these constructs simultaneously remain limited. Therefore, investigating the relationships among these variables, along with the role of psychological resilience, is of considerable importance. In light of these considerations, the present study sought to investigate the relationships among diabetes-specific disordered eating behaviors, social anxiety, social appearance anxiety, and psychological resilience in adolescents with type 1 diabetes.

Building on this framework, the present study aimed to address the following research questions:

1. What are the levels of diabetes-specific disordered eating behaviors, social anxiety, social appearance anxiety, and psychological resilience among adolescents with T1D?
2. Are there significant differences in diabetes-specific disordered eating behaviors, social anxiety, social appearance anxiety, and psychological resilience according to adolescents’ descriptive characteristics?
3. What are the correlations among diabetes-specific disordered eating behaviors, social anxiety, social appearance anxiety, and psychological resilience in adolescents with T1D?

Drawing on the existing literature, the following hypotheses were formulated:

**H1.**  *Higher levels of diabetes-specific disordered eating behaviors are anticipated to be associated with greater social anxiety and social appearance anxiety.*

**H2.**  *Higher psychological resilience is expected to be linked to lower levels of diabetes-specific disordered eating behaviors.*

**H3.**  *Social anxiety is expected to be positively related to social appearance anxiety.*

**H4.**  *Psychological resilience is expected to be inversely related to both social anxiety and social appearance anxiety.*

## 2. Materials and Methods

### 2.1. Study Design

The study employed a cross-sectional and correlational design with descriptive components and was carried out in accordance with the STROBE guidelines [17].

### 2.2. Participants and Procedure

The study population consisted of 370 adolescents diagnosed with T1D (ICD-10 codes E10.0–E10.9) who were registered and followed up at the pediatric endocrinology outpatient clinic of a training and research hospital between 25 September and 30 November 2024 (*N* = 370). The study sample comprised 176 adolescents who met the inclusion criteria, agreed to participate, and completed all study forms.

Sample size estimation was performed using G*Power version 3.1.9.7 (Heinrich Heine University Düsseldorf, Düsseldorf, Germany) [18]. Based on Cohen’s criteria for a medium effect size (f^2^ = 0.25) [19], with a 95% confidence level and a 5% margin of error, the minimum required sample size was calculated as 148 participants. To account for potential data loss, this number was increased by 10% [20], resulting in a target sample of at least 163 individuals. The study was ultimately completed with 176 adolescents who met the inclusion criteria, consented to participate, and provided complete data (*n* = 176).

#### 2.2.1. Inclusion Criteria

Participants were included if they met the following inclusion criterion:

1. Participants with a diagnosis of T1D defined by ICD-10 criteria were eligible for inclusion (E10.0–E10.9) [21],
2. Were adolescents aged 10–19 years [22],
3. Voluntarily agreed to participate,
4. Were literate,
5. Completed the data collection forms accurately, and
6. Had parental consent for participation.

#### 2.2.2. Exclusion Criteria

Individuals were excluded from the study if they met any of the following conditions:

1. Withdrew from the study after completing the forms,
2. Provided incomplete or invalid responses,
3. Had any diagnosis other than T1D,
4. Were illiterate,
5. Did not consent to participate.

### 2.3. Data Collection Instruments

Data collection involved a Descriptive Information Form and a set of validated instruments, including measures of diabetes-specific disordered eating (DEPS-R), social anxiety (SAS-A), social appearance anxiety (SAAS), and psychological resilience (CYRM-12).

#### 2.3.1. Descriptive Information Form

The Descriptive Information Form was developed by the researchers in line with the relevant literature [7,13,23] and consisted of five items capturing participants’ age, gender, educational level, duration of illness, height, and weight.

#### 2.3.2. Diabetes Eating Problem Survey-Revised (DEPS-R)

The Diabetes Eating Problem Survey–Revised (DEPS-R) is a self-report measure consisting of 16 items, originally developed by Markowitz et al. (2010) to evaluate diabetes-specific disordered eating behaviors [24]. Its Turkish validity and reliability for children and adolescents with T1D were established by Atik Altınok et al. (2017) [23]. Responses are rated on a 6-point Likert scale ranging from 0 (“never”) to 5 (“always”), yielding total scores between 0 and 80. Scores of 20 or higher are considered indicative of elevated risk for diabetes-specific disordered eating behaviors. The internal consistency of the scale was reported as α = 0.84 in the Turkish validation study [23] and was found to be α = 0.90 in the present study.

#### 2.3.3. Social Anxiety Scale for Adolescents (SAS-A)

The Social Anxiety Scale for Adolescents (SAS-A) was initially developed by La Greca et al. [25] and later revised for adolescent populations by La Greca and Lopez. Its Turkish validity and reliability were established by Aydın and Tekinsav Sütçü (2007) [26]. The instrument includes 22 items, of which 18 contribute to the total score, and responses are recorded on a 5-point Likert scale. Possible scores range from 18 to 90, with higher values reflecting greater levels of social anxiety. The internal consistency coefficient was reported as 0.88 for the Turkish version and was found to be 0.92 in the present study.

#### 2.3.4. Social Appearance Anxiety Scale (SAAS)

The Social Appearance Anxiety Scale (SAAS), developed by Hart et al. (2008) [27], is designed to evaluate anxiety related to physical appearance. Its Turkish adaptation was carried out by Doğan (2010) [28]. The scale includes 16 items rated on a 5-point Likert scale, with total scores ranging from 16 to 80; higher scores reflect greater levels of appearance-related anxiety. The internal consistency coefficient was reported as 0.93 in the Turkish validation study and was calculated as 0.92 in the present study.

#### 2.3.5. Child and Youth Resilience Measure (CYRM-12)

The Child and Youth Resilience Measure (CYRM-12) was originally developed by Liebenberg et al. (2012) [29] and later adapted into a shorter 12-item form [30]. Its Turkish validity and reliability were established by Arslan (2015) [31]. Responses are provided on a 5-point Likert scale, with total scores ranging from 12 to 60; higher scores indicate greater psychological resilience. The internal consistency coefficient was reported as 0.91 in the Turkish study and was found to be 0.94 in the present stud.

### 2.4. Data Collection

Data collection was carried out from 25 September to 30 November 2024, using in-person interviews conducted by the researcher. These interviews took place in a private room within the pediatric endocrinology outpatient clinic to ensure confidentiality. All procedures were implemented in a standardized manner and scheduled at times convenient for the participants, without interfering with routine clinical services. Completing the questionnaire required approximately 10 min.

#### Anthropometric Measurements

Anthropometric measurements were obtained using standardized procedures. Height was measured to the nearest 0.1 cm with a wall-mounted stadiometer, with participants standing barefoot, heels together, and positioned in the Frankfurt plane. Body weight was assessed to the nearest 0.1 kg using a calibrated digital scale, with participants wearing light clothing and no shoes. Body mass index (BMI) was calculated as weight in kilograms divided by the square of height in meters (kg/m^2^). Although age- and sex-specific BMI percentiles are typically recommended for pediatric populations, absolute BMI values were used in this study. However, WHO BMI-for-age reference standards for children and adolescents were considered when interpreting the results [32].

### 2.5. Data Analysis

Statistical analyses were performed using IBM SPSS Statistics version 25.0 (SPSS Inc., Chicago, IL, USA). Data distribution was evaluated based on skewness and kurtosis values, which fell within the acceptable range of −3 to +3, indicating suitability for parametric testing. Descriptive statistics, including frequencies, percentages, means, standard deviations, and minimum–maximum values, were calculated. Group comparisons were conducted using independent samples *t*-tests and one-way ANOVA, followed by Tukey post hoc tests where appropriate. Relationships among variables were examined using Pearson correlation analysis. Furthermore, multivariate linear regression analyses were performed to determine the independent associations between study variables. Statistical significance was defined as *p* < 0.05. Internal consistency was evaluated using Cronbach’s alpha coefficients [33,34].

### 2.6. Ethical Considerations

Ethical approval was obtained from the ethics committee of a public university prior to the commencement of the study (Date: 3 July 2024; Decision No: 240089-90). Institutional authorization and permission to use the study instruments were also secured. Participation was voluntary, and eligible adolescents were informed about the study using the Informed Consent Form, Child Assent Form, and Parental Consent Form. Only those who provided consent were included.

All data were collected and stored in accordance with confidentiality principles. Participants were informed that their involvement was entirely voluntary, that no personally identifiable information would be recorded, and that they could withdraw from the study at any stage without consequence. All information obtained was kept confidential, and participants were treated equally throughout the research process. The study was conducted in compliance with established ethical standards, including the principles outlined in the World Medical Association’s Declaration of Helsinki (2024/75 General Assembly).

## 3. Results

Participants’ descriptive characteristics are summarized in Table 1. The mean age of adolescents with type 1 diabetes (T1D) was 14.16 ± 2.73 years. Slightly over half of the sample were male (51.1%), 54.0% were enrolled in grades 5–8, and 63.1% had been living with T1D for more than three years. In addition, 66.5% of the participants had a body mass index (BMI) within the normal range, and 85.8% were categorized as being at high risk for diabetes-specific disordered eating behaviors (Table 1).

The mean scores obtained from the study scales are summarized in Table 2. The average scores were 36.06 ± 15.26 for DEPS-R, 58.86 ± 12.90 for SAS-A, 48.82 ± 12.09 for SAAS, and 35.17 ± 10.61 for CYRM-12.

Table 3 summarizes the differences in scale scores according to participants’ descriptive characteristics. Higher DEPS-R scores were observed among adolescents aged 15–19 years (38.84 ± 16.65), those diagnosed with T1D for three years or longer (37.82 ± 16.57), and those with a high risk of diabetes-specific disordered eating behaviors (40.09 ± 12.25). A significant difference in DEPS-R scores was observed across BMI categories (*p* < 0.05), with higher scores noted among overweight and obese participants compared to their underweight and normal-weight peers. However, no statistically significant difference was found between female (35.12 ± 16.34) and male (36.91 ± 14.19) participants in terms of DEPS-R scores (t = −0.779, *p* = 0.437).

Participants were additionally classified into high-risk (DEPS-R ≥ 20) and low-risk (DEPS-R < 20) groups. Adolescents in the high-risk group demonstrated significantly higher levels of social anxiety (60.26 ± 11.97 vs. 50.44 ± 15.20; t = 3.647, *p* < 0.001) and social appearance anxiety (49.69 ± 12.06 vs. 43.56 ± 11.11; t = 2.379, *p* = 0.018) compared with those in the low-risk group. Conversely, psychological resilience scores were significantly lower among high-risk participants (26.72 ± 11.87 vs. 36.56 ± 9.74; t = 4.531, *p* < 0.001). Furthermore, both social anxiety and social appearance anxiety differed significantly across BMI categories (*p* < 0.05), with higher scores observed in overweight and obese adolescents. All reported differences were statistically significant (Table 3).

Pearson correlation analysis indicated significant relationships among diabetes-specific disordered eating behaviors, social anxiety, social appearance anxiety, and psychological resilience in adolescents with T1D (Table 4). DEPS-R scores showed moderate positive correlations with SAS-A (r = 0.51) and SAAS (r = 0.43), and a moderate negative correlation with psychological resilience (CYRM-12) (r = −0.48). Social anxiety scores (SAS-A) were strongly positively correlated with social appearance anxiety (SAAS) (r = 0.59) and moderately negatively correlated with CYRM-12 (r = −0.52). Likewise, SAAS scores demonstrated a moderate inverse association with CYRM-12 (r = −0.56). All reported correlations were statistically significant.

Building on the correlation findings, multivariate linear regression analyses were performed to evaluate the independent relationships among the study variables (Table 5). For diabetes-specific disordered eating behaviors (DEPS-R), the model was statistically significant (F = 23.630, *p* < 0.001) and accounted for 29.2% of the variance (R^2^ = 0.292). Social anxiety showed a significant positive association with DEPS-R scores (β = 0.359, *p* < 0.001), whereas social appearance anxiety (β = 0.163, *p* = 0.059) and psychological resilience (β = −0.103, *p* = 0.200) were not significant predictors. For social anxiety, the regression model was also significant (F = 48.476, *p* < 0.001), explaining 45.8% of the variance (R^2^ = 0.458). Both disordered eating behaviors (β = 0.274, *p* < 0.001) and social appearance anxiety (β = 0.353, *p* < 0.001) were positively associated with social anxiety, while psychological resilience was inversely related (β = −0.214, *p* = 0.002). In the model for social appearance anxiety, a significant result was observed (F = 46.564, *p* < 0.001), accounting for 44.8% of the variance (R^2^ = 0.448). Social anxiety emerged as a positive predictor (β = 0.359, *p* < 0.001), whereas psychological resilience was negatively associated (β = −0.325, *p* < 0.001). Disordered eating behaviors did not demonstrate an independent association (β = 0.127, *p* = 0.059). Finally, the model for psychological resilience was significant (F = 33.630, *p* < 0.001) and explained 37.0% of the variance (R^2^ = 0.370). Both social anxiety (β = −0.249, *p* = 0.002) and social appearance anxiety (β = −0.371, *p* < 0.001) were negatively associated with resilience, whereas disordered eating behaviors were not independently related (β = −0.092, *p* = 0.200).

## 4. Discussion

This study explored the relationships among diabetes-specific disordered eating behaviors, social anxiety, social appearance anxiety, and psychological resilience in adolescents with type 1 diabetes (T1D). The results suggest that, in addition to physiological demands, adolescents with T1D are also exposed to substantial psychosocial challenges.

In this study, diabetes-specific disordered eating behaviors among adolescents with T1D were evaluated using the DEPS-R, with scores of ≥20 indicating elevated risk in accordance with prior validation research [23]. A notable finding was the high proportion of participants (85.8%) classified as being at risk. This estimate exceeds those reported in earlier studies, where prevalence rates generally range between approximately 11.2% and 52.8% [35,36].

However, similar trends indicating increased risk in this population have also been reported in the literature. For example, prevalence rates of 47.9% and 45.7% have been documented among adolescents with T1D [37,38], and other studies have emphasized the increased vulnerability of this group to disordered eating behaviors [39]. This finding is supported by a systematic review conducted by Dean et al., which reported an increased risk of eating disorders among individuals with T1D [10]. Overall, our findings are consistent with the existing literature and suggest that adolescents with T1D represent a population at elevated risk for diabetes-specific disordered eating behaviors.

Several factors may help explain the relatively high prevalence observed in the present study. First, the use of the DEPS-R cut-off score (≥20), which is widely accepted for identifying individuals at high risk [23], may have influenced prevalence estimates. Second, the clinical characteristics of the sample, including adolescents receiving care in a specialized setting, may reflect a population with a higher disease burden or greater psychosocial vulnerability. Third, reliance on self-reported measures may have introduced response-related biases. In addition, differences across studies may be explained by variations in sample characteristics, cultural and contextual factors, and methodological approaches [36].

Prior studies have indicated that elevated DEPS-R scores are linked to factors such as older age, higher body mass index (BMI), and poorer glycemic control [40]. Consistent with this, Köprülü et al. (2025) identified significant positive relationships between BMI and DEPS-R scores, suggesting that greater body weight may increase the likelihood of disordered eating behaviors among adolescents with T1D [41]. The demands of managing T1D—including ongoing dietary regulation, calorie monitoring, and heightened attention to body weight—may facilitate the emergence of maladaptive eating patterns. This susceptibility may be further intensified during adolescence, a developmental period when concerns related to body image become more prominent [23]. Taken together, these findings suggest that both clinical and psychosocial factors contribute to the understanding of diabetes-specific disordered eating behaviors. Overall, the results reinforce the view that adolescents with T1D constitute a population at elevated risk, while also emphasizing the need to interpret prevalence estimates in light of methodological considerations and sample characteristics.

The findings of the present study suggest that the likelihood of diabetes-specific disordered eating behaviors increases with advancing age and longer duration of illness. In line with this, Alguwaihes et al. (2025) reported that the risk of disordered eating behaviors tends to rise with age among adolescents with T1D [36]. As adolescents grow older, they may experience greater concerns about body weight and are more likely to adopt unhealthy eating habits [42,43]. This may reflect the cumulative psychological burden associated with long-term disease management. Consistent with the literature, longer duration of diabetes may be associated with greater challenges in coping, which may be reflected in maladaptive eating behaviors [39,43]. Moreover, the observed relationship between higher BMI and an increased risk of disordered eating behaviors underscores the pivotal role of body image in this association [38].

Participants demonstrated elevated levels of both social anxiety and social appearance anxiety. Lifestyle demands associated with T1D, perceived differences in peer relationships, and challenges in self-expression within social contexts may contribute to elevated anxiety levels [9,44]. Moreover, the responsibilities of managing a chronic illness and engaging in visible health-related behaviors (e.g., insulin administration and dietary regulation) may increase adolescents’ sensitivity to social evaluation. Adolescents with greater levels of diabetes-specific disordered eating behaviors tended to report higher levels of both social anxiety and social appearance anxiety, indicating a potential interconnection among these variables. Previous studies similarly indicate that individuals with poorer metabolic control are more likely to experience psychiatric symptoms [38,45]. Moreover, increased levels of disordered eating behaviors have been linked to reduced self-esteem and more pronounced interpersonal difficulties [38,43].

A significant positive relationship was observed between social anxiety and social appearance anxiety, reinforcing the view that concerns about body image are closely related to fears of social evaluation during adolescence. Adolescents’ perceptions of their physical appearance are closely related to their need for social acceptance, which may contribute to increased levels of social anxiety [46].

Although psychological resilience showed an association with diabetes-specific disordered eating behaviors in the correlation analysis, this relationship was no longer significant after adjusting for other variables in the regression model. In contrast, psychological resilience showed consistent negative associations with both social anxiety and social appearance anxiety. The regression model accounted for a moderate proportion of the variance in diabetes-specific disordered eating behaviors (R^2^ = 0.29), indicating a moderate level of explanatory capacity. Among the predictors included in the model, social anxiety was the only variable that remained a statistically significant independent factor associated with diabetes-specific disordered eating behaviors (β = 0.36, *p* < 0.001).

These results indicate that psychological resilience is more strongly linked to anxiety-related outcomes than to diabetes-specific disordered eating behaviors when evaluated within a multivariate framework. In line with prior studies, individuals with higher resilience tend to manage stress more effectively and experience lower levels of psychological distress [13,14]. Research involving children and adolescents with T1D has likewise reported inverse relationships between resilience and anxiety [35]. Moreover, greater resilience has been associated with stronger social connections and improved quality of life [14], underscoring its broader role in psychosocial functioning.

Overall, the results suggest that diabetes-specific disordered eating behaviors, social anxiety, and social appearance anxiety are closely interconnected among adolescents with T1D, while psychological resilience appears to play a more prominent role in relation to social anxiety-related outcomes. These findings underscore the importance of incorporating psychosocial evaluation and interventions into routine diabetes care. Multidisciplinary strategies, including psychological counseling, psychoeducation, and resilience-enhancing interventions, may provide meaningful support for this population.

This study has several limitations. First, the single-center design may restrict the generalizability of the results. Second, reliance on self-reported measures may introduce the possibility of response bias. In addition, the absence of clinical variables such as Tanner stage, mode of insulin therapy, insulin dose, and glycemic control may have limited the ability to account for potential confounding factors in adolescents with T1D. Furthermore, BMI was evaluated using absolute values rather than age- and sex-specific percentiles, which may reduce its accuracy in adolescent populations. The lack of a healthy control group also limits the ability to compare the prevalence of diabetes-specific disordered eating behaviors with that of the general adolescent population. Finally, the cross-sectional design of the study does not allow for causal interpretations, and the direction of the observed relationships cannot be established. These constraints should be considered when interpreting the findings, as they may affect the generalizability of the results and limit conclusions about the direction of these associations.

Despite these limitations, the study has several notable strengths. It provides a comprehensive assessment of diabetes-specific disordered eating behaviors, social anxiety, social appearance anxiety, and psychological resilience within a single clinical sample of adolescents with T1D. The use of validated measurement tools strengthens the reliability of the findings, while the inclusion of multiple psychosocial constructs enables a more comprehensive understanding of the relationships among these variables. In addition, the use of multivariate analyses strengthens the analytical rigor of the study by enabling the examination of independent associations among variables. The sample size is also adequate for the analyses performed, supporting the robustness of the findings.

## 5. Conclusions

The findings of this study indicate that a large proportion of adolescents with type 1 diabetes (T1D) (85.8%) are at elevated risk for diabetes-specific disordered eating behaviors. These adolescents also exhibited elevated levels of social anxiety and social appearance anxiety, along with moderate levels of psychological resilience. Older age (15–19 years), longer disease duration (≥3 years), and higher BMI (overweight and obese) were associated with higher disordered eating scores. Adolescents with higher disordered eating risk exhibited significantly greater social anxiety and social appearance anxiety, whereas male adolescents and those with lower risk demonstrated higher psychological resilience. Correlation analyses revealed moderate positive associations between disordered eating risk and both social anxiety and social appearance anxiety, and negative associations between psychological resilience and these variables. Multivariate regression analyses showed that social anxiety remained an independent predictor of disordered eating behaviors, whereas psychological resilience did not demonstrate an independent association after controlling for other variables. In contrast, psychological resilience was inversely related to both social anxiety and social appearance anxiety. These findings highlight the complex interplay among psychosocial variables in adolescents with T1D and underscore the importance of addressing social anxiety and social appearance concerns in this population.

Based on these findings, routine screening for diabetes-specific disordered eating behaviors and social anxiety-related symptoms should be systematically integrated into the clinical follow-up of adolescents with T1D. Interventions targeting social anxiety and social appearance concerns should be implemented, alongside approaches aimed at strengthening psychological resilience to support overall psychosocial well-being. A multidisciplinary care model involving endocrinologists, mental health professionals, dietitians, diabetes nurses, and psychiatric nurses is recommended to ensure comprehensive and holistic management. Future research should include clinically relevant factors, such as pubertal stage, characteristics of insulin therapy, and glycemic control, to achieve a more comprehensive understanding of variables associated with diabetes-specific disordered eating behaviors. Longitudinal designs are also recommended to better clarify the direction of the observed associations. It is also important for future studies to include healthy comparison groups to better contextualize these findings. Integrating both psychosocial and clinical parameters may further enhance understanding of diabetes-specific disordered eating behaviors in adolescents with type 1 diabetes.

## Figures and Tables

**Table 1:** Descriptive characteristics of adolescents with type 1 diabetes.

Descriptive Characteristics	Groups	n	%
**Age** Mean = 14.16 ± 2.73 (Min = 10, Max = 19)	10–14 years	97	55.1
	15–19 years	79	44.9
**Gender**	Female	86	48.9
	Male	90	51.1
**Grade Level**	Grades 5–8	95	54.0
	Grades 9–12	81	46.0
**Duration of Type 1 Diabetes Diagnosis**	1–3 years	65	36.9
	≥3 years	111	63.1
**BMI** Mean = 21.66 ± 3.98 (Min = 11.80, Max = 34.6)	Underweight (BMI ≤ 18.4)	37	21.0
	Normal (BMI: 18.5–24.9)	117	66.5
	Overweight (BMI: 25.0–29.9)	14	8.0
	Obese (BMI: 30.0 and above)	8	4.5
**DEPS-R** Mean = 36.03 ± 15.26 (Min = 0, Max = 71)	High risk of diabetes-specific disordered eating behaviors (DEPS-R ≥ 20)	151	85.80
	Low risk of diabetes-specific disordered eating behaviors (DEPS-R < 20)	25	14.20
**Total**		176	100

Notes: BMI: Body Mass Index; DEPS-R: Diabetes Eating Problem Survey-Revised.

**Table 2:** Mean total scores of DEPS-R, SAS-A, SAAS, and CYRM-12 among adolescents with type 1 diabetes.

Scales (n = 176)	Min.	Max.	Mean ± SD
DEPS-R	0	71	36.03 ± 15.26
SAS-A	23	90	58.86 ± 12.90
SAAS	20	77	48.82 ± 12.09
CYRM-12	12	57	35.17 ± 10.61

Notes: DEPS-R: Diabetes Eating Problem Survey-Revised; SAS-A: Social Anxiety Scale for Adolescents; SAAS: Social Appearance Anxiety Scale; CYRM-12: Child and Youth Resilience Measure.

**Table 3:** Differences in DEPS-R, SAS-A, SAAS, and CYRM-12 scores by descriptive characteristics of adolescents with type 1 diabetes.

Descriptive Characteristics	DEPS-R Mean ± SD Test and *p*	SAS-A Mean ± SD Test and *p*	SAAS Mean ± SD Test and *p*	CYRM-12 Mean ± SD Test and *p*
**Age**				
10–14 years	33.75 ± 13.69	58.33 ± 13.06	48.34 ± 11.74	34.90 ± 10.56
15–19 years	38.84 ± 16.65	59.52 ± 12.75	49.41 ± 12.55	35.45 ± 10.73
	t = −2.223, *p* = 0.028	t = −0.607, *p* = 0.545	t = −0.580, *p* = 0.563	t = −0.355, *p* = 0.722
**Gender**				
Female	35.12 ± 16.34	57.69 ± 13.38	48.80 ± 12.29	32.69 ± 10.76
Male	36.91± 14.19	59.99 ± 12.39	48.83 ± 11.96	37.53 ± 9.96
	t = −0.779, *p* = 0.437	t = −1.185, *p* = 0.237	t = −0.017, *p* = 0.986	t = −3.140, *p* = 0.002
**Duration of Type 1 Diabetes Diagnosis**				
1–3 years	32.98 ± 12.24	57.12 ± 10.06	48.06 ± 9.70	35.42 ± 9.17
≥3 years	37.82 ± 16.57	59.88 ± 14.25	49.26 ± 13.31	35.02 ± 11.40
	t= −2.047, *p* = 0.042	t = −1.373, *p* = 0.171	t = −0.634, *p* = 0.527	t = −0.239, *p* = 0.811
**BMI**				
Underweight ^1^	31.76 ± 14.54	58.51 ± 11.55	50.08 ± 11.77	34.51 ± 11.75
Normal ^2^	34.56 ± 13.75	56.18 ± 10.87	46.12 ± 9.37	34.57 ± 10.14
Overweigh ^3^	48.50 ± 18.03	66.92 ± 16.77	56.00 ± 17.79	37.00 ± 9.29
Obese ^4^	53.25 ± 14.03	81.75 ± 6.86	71.75 ± 5.65	44.63 ± 11.29
	F = 7.044, *p* = 0.001	F = 12.857, *p* = 0.001	F = 13.537, *p* = 0.001	F = 2.016, *p* = 0.194
Post hoc=	3, 4 > 1, 2	3, 4 > 1, 2	3, 4 > 1, 2	
**DEPS-R**				
High risk of diabetes-specific disordered eating behaviors (DEPS-R ≥ 20)	40.09 ± 12.25	60.26 ± 11.97	49.69 ± 12.06	26.72 ± 11.87
Low risk of diabetes-specific disordered eating behaviors (DEPS-R < 20)	11.52 ± 5.44	50.44 ± 15.20	43.56 ± 11.11	36.56 ± 9.74
	t = 11.453, *p* = 0.001	t = 3.647, *p* = 0.001	t = 2.379, *p* = 0.018	t = 4.531, *p* = 0.001

Notes: SD: Standard deviation; F: Analysis of variance (ANOVA); t: Independent samples *t*-test; Post hoc: Tukey’s test; DEPS-R: Diabetes Eating Problem Survey-Revised; SAS-A: Social Anxiety Scale for Adolescents; SAAS: Social Appearance Anxiety Scale; CYRM-12: Child and Youth Resilience Measure; BMI: Body Mass Index; Superscript numbers indicate statistically significant differences between groups according to post-hoc analyses.

**Table 4:** Correlations among DEPS-R, SAS-A, SAAS, and CYRM-12 scores in adolescents with Type 1 Diabetes.

Scales	1	2	3	4
1. DEPS-R	1			
2. SAS-A	0.508 *	1		
3. SAAS	0.432 *	0.591 *	1	
4. CYRM-12	−0.479 *	−0.515 *	−0.558 *	1

Notes: DEPS-R: Diabetes Eating Problem Survey-Revised; SAS-A: Social Anxiety Scale for Adolescents; SAAS: Social Appearance Anxiety Scale; CYRM-12: Child and Youth Resilience Measure. * *p* < 0.05; all correlations are significant at *p* < 0.001.

**Table 5:** Multivariate regression models examining predictors of DEPS-R, SAS-A, SAAS, and CYRM-12 scores in adolescents with type 1 diabetes.

Predictor	Model 1: DEPS-R β	*p*	Model 2: SAS-A β	*p*	Model 3: SAAS β	*p*	Model 4: CYRM-12 β	*p*
DEPS-R	—	—	0.274	<0.001	0.127	0.059	−0.092	0.200
SAS-A	0.359	<0.001	—	—	0.359	<0.001	−0.249	0.002
SAAS	0.163	0.059	0.353	<0.001	—	—	−0.371	<0.001
CYRM-12 (Resilience)	−0.103	0.200	−0.214	0.002	−0.325	<0.001	—	—
R^2^	0.292	—	0.458	—	0.448	—	0.370	—
F	23.630	<0.001	48.476	<0.001	46.564	<0.001	33.630	<0.001

Notes: DEPS-R: Diabetes Eating Problem Survey-Revised; SAS-A: Social Anxiety Scale for Adolescents; SAAS: Social Appearance Anxiety Scale; CYRM-12: Child and Youth Resilience Measure; β = standardized regression coefficient; R^2^ = coefficient of determination; F = F statistic.

## Data Availability

The data presented in this study is available on request from the corresponding author. The data is not publicly available due to privacy and ethical restrictions, as it contains sensitive personal and health-related information.

## Abbreviations

The following abbreviations are used in this manuscript:

DM	Diabetes Mellitus
T1D	Type 1 Diabetes
BMI	Body Mass Index
DEPS-R	Diabetes Eating Problem Survey-Revised
SAS-A	Social Anxiety Scale for Adolescents
SAAS	Social Appearance Anxiety Scale
CYRM-12	Child and Youth Resilience Measure
DSM-5	The Diagnostic and Statistical Manual of Mental Disorders-5
ICD-10	International Classification of Disease-10
WHO	World Health Organization
